# Projection Stereolithographic Fabrication of Human Adipose Stem Cell-Incorporated Biodegradable Scaffolds for Cartilage Tissue Engineering

**DOI:** 10.3389/fbioe.2015.00115

**Published:** 2015-08-18

**Authors:** Aaron X. Sun, Hang Lin, Angela M. Beck, Evan J. Kilroy, Rocky S. Tuan

**Affiliations:** ^1^Center for Cellular and Molecular Engineering, Department of Orthopaedic Surgery, University of Pittsburgh School of Medicine, Pittsburgh, PA, USA; ^2^Medical Scientist Training Program, University of Pittsburgh School of Medicine, Pittsburgh, PA, USA; ^3^Department of Bioengineering, University of Pittsburgh Swanson School of Engineering, Pittsburgh, PA, USA

**Keywords:** adipose stem cells, cartilage tissue engineering, PDLLA-PEG, projection stereolithography, live cell-scaffold fabrication

## Abstract

The poor self-healing ability of cartilage necessitates the development of methods for cartilage regeneration. Scaffold construction with live stem cell incorporation and subsequent differentiation presents a promising route. Projection stereolithography (PSL) offers high resolution and processing speed as well as the ability to fabricate scaffolds that precisely fit the anatomy of cartilage defects using medical imaging as the design template. We report here the use of a visible-light-based PSL (VL-PSL) system to encapsulate human adipose-derived stem cells (hASCs) into a biodegradable polymer [poly-d,l-lactic acid/polyethylene glycol/poly-d,l-lactic acid (PDLLA-PEG)]/hyaluronic acid (HA) matrix to produce live cell constructs with customized architectures. After fabrication, hASCs showed high viability (84%) and were uniformly distributed throughout the constructs, which possessed high mechanical properties with a compressive modulus of 780 kPa. The hASC-seeded constructs were then cultured in control or TGF-β3-containing chondrogenic medium for up to 28 days. In chondrogenic medium-treated group (TGF-β3 group), hASCs maintained 77% viability and expressed chondrogenic genes Sox9, collagen type II, and aggrecan at 11, 232, and 2.29 × 10^5^ fold increases, respectively compared to levels at day 0 in non-chondrogenic medium. The TGF-β3 group also produced a collagen type II and glycosaminoglycan-rich extracellular matrix, detected by immunohistochemistry, Alcian blue staining, and Safranin O staining suggesting robust chondrogenesis within the scaffold. Without chondroinductive addition (Control group), cell viability decreased with time (65% at 28 days) and showed poor cartilage matrix deposition. After 28 days, mechanical strength of the TGF-β3 group remained high at 240 kPa. Thus, the PSL and PDLLA-PEG/HA-based fabrication method using adult stem cells is a promising approach in producing mechanically competent engineered cartilage for joint cartilage resurfacing.

## Introduction

Cartilage damaged by trauma, disease, or aging demonstrates very limited capabilities for self-regeneration and ultimately results in osteoarthritis (OA) (Tuan et al., 2013). Given the high prevalence of OA in the United States (27 million affected) (Zhang and Jordan, 2010), which is projected to increase due to population aging as well as the obesity epidemic, methods toward managing and treating these cartilage defects are critical. While there exist procedures to treat these defects, such as microfracture and osteochondral grafting, they either finally lead to the formation of fibrocartilage or are limited by tissue availability (Moriya et al., 2007; Gigante et al., 2011; Ye et al., 2013). In addition, allografting bone and cartilage have the potential risk of infection and disease transmission. Severe cartilage defects ultimately require total joint arthroplasty to reduce pain and improve mobility, but this involves a major surgery and ends the biological life of cartilage (Moran and Horton, 2000; Nashi et al., 2014). As such, a regenerative approach that can restore the native properties of cartilage represents an attractive alternative.

Recently, regenerative medicine has garnered high interest, which involves the development of cartilage-like constructs through the use of cells, growth factors, scaffolds, and combinations (Tuan et al., 2013; Demoor et al., 2014). For example, autologous chondrocyte implantation (ACI) and matrix-induced ACI (MACI) are popular procedures that harvest and expand chondrocytes *in vitro* from the patient’s own tissue, which are then grafted into the cartilage defect site with or without accompanying extracellular matrix (ECM). However, this source of healthy chondrocytes is limited and requires several weeks of cell culturing to obtain adequate cell numbers for transplantation, which results in chondrocyte dedifferentiation (Kuo et al., 2006). Thus, adult tissue-derived stem/progenitor cells, such as mesenchymal stem cells (MSCs), that have been shown to have the ability to differentiate into a variety of cell lines including chondrocytes (Pittenger et al., 1999) offer a promising substitute for the primary chondrocytes. In particular, adipose tissue-derived MSCs (ASCs) have attracted recent attention because they are isolated in higher quantities than stem cells found from other sources, such as bone marrow, and are obtainable through minimally invasive procedures, thus offering the advantage of reducing or even eliminating *in vitro* expansion to allow point-of-care application (Roux et al., 2013). In addition, ASCs have been shown to be beneficial in cartilage healing, including reducing pain and improving function for aging patients with knee OA after intra-articular injection (Koh et al., 2013).

A key component in cartilage tissue engineering is a biomaterial scaffold to deliver the candidate cells, such as MSCs, to the defect site and to also temporarily fill the defect to facilitate cell growth. In addition to biocompatibility, the ideal scaffold for cartilage tissue engineering should possess viscoelastic hydrogel-like characteristics that mimic the mechanical properties and functions of native cartilage. Optimally, the biomaterial will also be biodegradable such that as the stem cells differentiate into chondrocytes and produce a cartilaginous ECM, the scaffold degrades and the newly secreted ECM remodels the construct into a cartilage-like tissue. In addition, precise fitting of the scaffold into the local structural geometry of the defect and the host tissue anatomy is critical for enhancing the repair process such that the absence of gaps will optimize integration between implants and native tissue and allow continuous load distribution (Da Silva et al., 2012). To date, many technologies have been developed in the fabrication of scaffolds with different geometry and internal architecture. Traditional technologies, such as solvent casting, particulate leaching, and electrospinning, do allow for limited control of structure, but they are not able to perform the fabrication of highly detailed structures on a patient by patient basis (Yang et al., 2001). In addition, these methods require the use of either organic solvents or conditions unfavorable for cell survival, thereby limiting their ability to seed cells directly within scaffolds (Yang et al., 2002; Derby, 2012).

Solid free-form fabrication (SFF) methods have been shown to offer the ability to control both the macrostructure as well as the microstructure of scaffolds. With the utilization of medical imaging and computer-aided design (CAD) model guided scaffold fabrication, SFF methods can create scaffolds with precise architectures (Bajaj et al., 2012). Different SFF methods have been applied in the fabrication of a variety of biomaterials, including laser sintering, stereolithography, fused deposition modeling, and 3D printing. Of these techniques, stereolithography is the most accurate and is based on light-induced photo-polymerization of derivatized monomers (Melchels et al., 2010). Projection stereolithography (PSL) in particular is a method that has been attracting increased interest due to its high fabrication rate and resolution. By utilizing a layer-by-layer-based image projection of defined thickness, fabrication times are drastically reduced from conventional stereolithography. Recently, in our laboratory, PSL using visible-light illumination (VL-PSL) has been applied in a one-step live cell-scaffold fabrication in which highly viable human adipose-derived stem cells (hASCs) were uniformly incorporated within polyethylene glycol diacrylate (PEGDA) scaffolds (Lin et al., 2013). While this procedure for the first time allows live cell-scaffold fabrication using VL-PSL, it is currently limited by the monomer that is used because PEG is not biodegradable and does not provide cell-binding ligands. In addition, Percoll was used to suspend the cells during the fabrication, which introduces additional non-native molecules into the final construct. The extent to which chondrogenesis of hASCs is supported in the PEG scaffold was also not investigated.

A number of properties are desirable for a biomaterial suitable for implantation for cartilage repair, including biodegradability, biocompatibility, strong compressive modulus, and presence of cell-binding ligands. Most importantly, the material must also be water-soluble in order to not interfere with cell survival during the photo-crosslinking scaffold fabrication process. We report here the identification of a novel hybrid matrix in which the synthetic polymer, poly-d,l-lactic acid/polyethylene glycol/poly-d,l-lactic acid (PDLLA-PEG), served as the structural component (Seck et al., 2010), and hyaluronic acid (HA) as the co-polymer to supply cell-binding ligands and to inhibit cell settlement during PSL fabrication owing to its viscous property. HA is a glycosaminoglycan (GAG) present in abundance in the cartilage ECM and synovial fluid and has been shown to promote hASC chondrogenesis through interaction with its surface receptor, CD44 (Wu et al., 2013; Chopra et al., 2014).

In this study, hASCs were suspended in a methacrylated PDLLA-PEG and HA (mPDLLA-PEG) solution and subjected to VL-PSL with different CAD architectures. The cell-seeded fabricated scaffolds were cultured in control medium or TGF-β3-containing chondrogenic medium for up to 4 weeks. Cell viability was examined at different time points and the progression of chondrogenesis of hASCs within the scaffolds was assessed by mechanical testing, real time reverse transcription polymerase chain reaction (RT-PCR) analysis of gene expression, and histological staining. Our results showed that the VL-PSL produced hybrid scaffolds precisely mimicked the CAD structure and maintained high cell viability during the fabrication process. In addition, mPDLLA-PEG/HA scaffolds supported efficient hASC chondrogenesis upon induction. Thus, the method described in this report represents a promising method for the development of personalized stem cell-based repair of articular cartilage defect.

## Materials and Methods

All chemicals were purchased from Sigma-Aldrich (St. Louis, MO, USA) unless otherwise stated.

### Human adipose stem cell isolation

Human adipose-derived stem cells were isolated from lipoaspirate with Institutional Review Board approval (University of Pittsburgh and University of Washington) using an automated cell isolation system from Tissue Genesis, Inc. (Honolulu, HI, USA). The isolated cell pellets were re-suspended in expansion medium (EM: DMEM-high glucose, 10% MSC-certified fetal bovine serum (FBS), 100 U/ml penicillin, 100 μg/ml streptomycin; Invitrogen, Carlsbad, CA, USA) and plated on tissue culture flask. After 3 days, the non-attached cells were washed out with Hank’s Balanced Salt Solution (HBSS). The medium was changed every 3 days. At 80% confluence, cells were detached with 0.25% trypsin in 1 mM EDTA (Invitrogen) and passaged. All experiments were performed with hASCs obtained at passage 3 (P3). hASCs used in this study were pooled from two patients (36- and 28-year females).

### Synthesis of methacrylated PDLLA-PEG and HA

Preparation of the mPDLLA-PEG was performed as described by Seck et al. (2010). Briefly, 50 g of PEG (4 kDa molecular weight) was placed into a 250 ml Erlenmeyer flask and subjected to 600 W microwave irradiation for 3 min. Subsequently, 3.5 g (2.80 ml) of stannous octoate [Sn(Oct)_2_] was added to the molten PEG followed by addition of 7.2 g poly-d,l-lactide. The mixture was subjected to 600 W microwave irradiation for 1 min. The initial PDLLA-PEG polymer was precipitated in 500 ml cold isopropanol, and was dried under vacuum for 2 days. Dry polymer was dissolved in 100 ml dichloromethane (DCM), followed by addition of three equivalents of triethylamine (TEA, ~5.25 ml) and three equivalents of methacrylic anhydride (MA, ~5.60 ml). The reaction mixture was covered with punctured Parafilm and allowed to stir at room temperature for 7 days. After completion of the reaction, the mixture was precipitated into diethyl ether. For further purification, the macromer was redissolved in minimal amounts of chloroform and reprecipitated in diethyl ether.

Methacrylated hyaluronic acid was prepared by reacting MA with sodium hyaluronate (research grade, MW ~700 kDa, Lifecore, Chaska, MN) (Chung et al., 2009).

### Synthesis of photoinitiator LAP

The visible-light sensitive initiator lithium phenyl-2,4,6-trimethylbenzoylphosphinate (LAP) was synthesized as described by Fairbanks et al. (2009).

### PSL fabrication of live cell constructs

The PSL apparatus was purchased from EnvisionTec (Perfactory Standard, Gladbeck, Germany) equipped with digital light processing (DLP) technology. Visible-light mode (Hg illumination utilizing a UV barrier filter) was used, with the curing depth of each layer set at 50 μm.

Solutions of polymer, LAP, and phenol red dye were prepared in 50 ml tubes. Thirty percent PDLLA-PEG was chosen because it not only maintained the fabrication fidelity of VL-PSL but also formed a scaffold with mechanical properties more similar to that of cartilage. HA was used at 0.5% concentration because it yielded sufficient specific gravity to suspend cells but did not compromise routine cell mixing by trituration, for example, by repeated pipetting. For instance, the preparation of mPDLLA-PEG (30% w/v), mHA (0.5% w/v), LAP (0.6% w/v), and phenol red (0.025% w/v) was carried out as follows: polymer (12 g mPDLLA-PEG and 0.2 g mHA) was placed in the 50 ml tube followed by slow addition of HBSS close to the 40 ml mark and subsequent addition of LAP (240 mg) and phenol red (10 mg). The solution was titrated to pH 7.4 with 10 N NaOH and adjusted to 40 ml using HBSS.

P3 hASCs were pelleted by centrifugation, and the supernatant was completely removed. The polymer solution prepared above was added on top of the pellets and mixed with cells thoroughly by gently pipetting up and down 20 times. The final hASC density was 4 × 10^6^ cells/ml. After the bubbles were removed by aspiration, the cell-polymer solution was immediately poured into the basement plate of the PSL device for printing with different 3D models as the template, using our recently described procedure (Lin et al., 2013).

The fabricated constructs were detached from the platform and washed three times to remove uncured polymer solution. The constructs were cultured in control medium [CM, DMEM with1% l-alanyl-l-glutamine (GlutaMAX), 55 μM sodium pyruvate, 1× antibiotic-antimycotic, and 1% insulin–transferrin–selenium (ITS) (Invitrogen, Carlsbad, CA, USA)] or chondrogenic medium [CGM, CM supplemented with 10 ng/ml transforming growth factor-β3 (TGF-β3; PeproTech, Rocky Hill, NJ, USA], 100 nM dexamethasone, 50 μM l-ascorbic acid 2-phosphate, and 23 μM l-proline) up to 28 days, which were designated as control or TGF-β3 group, respectively.

### Degradation test

Fabricated scaffolds (5 mm diameter and 2 mm height) produced as described above but without cells were immersed in 5 ml HBSS and maintained in cell culture incubator at 37°C. HBSS was changed every 3 days. Since our ultimate goal was to apply the engineered tissue constructs for articular cartilage repair *in vivo*, their functional characteristics, particularly in terms of mechanical property, were of critical importance. Therefore, the degradation of polymer was estimated by measuring the mechanical property of scaffolds at different times.

Mechanical testing of scaffolds was conducted with a mechanical tester (Bose Electroforce model 3230 Series II). Briefly, the cylindrical scaffolds were placed between the compressive motor and load cell and subjected to 10% compression (0.2 mm) at 0.01 mm/s. The stress–strain curve was then plotted, and the linear area was used to calculate the compressive modulus of scaffolds.

### Live/dead staining

At various time points post-fabrication, cell viability was assessed with the live/dead viability/cytotoxicity kit (Invitrogen) as examined by epifluorescence microscopy following the product manual. The percentage of live cells was calculated as the number of green-staining cells divided by the total number of cells (green and red staining cells).

### MTS assay

On days 0 and 28, cell metabolic activity was assessed with CellTiter 96^®^ AQueous One Solution Cell Proliferation Assay [3- (4,5-dimethylthiazol-2-yl)-5-(3-carboxymethoxyphenyl)-2-(4-su lfophenyl)-2H-tetrazolium, inner salt; MTS, Promega, Madison, WI, USA]. The constructs with cells were cultured with MTS solution for 4 h, and absorbance at 492 nm was measured using a microplate reader (BioTek, Winooski, VT, USA).

### Analysis of gene expression by real-time RT-PCR

Total RNA of the cells within the constructs was isolated using TRIZOL reagent (Invitrogen) and purified using RNeasy Plus Mini Kit (Qiagen, Germantown, MD, USA). Reverse transcription reactions were performed using SuperScript^®^ VILO™ cDNA Synthesis Kit (Invitrogen) according to manufacturer’s manual. Real-time PCR was performed using the SYBR Green Reaction Mix (Applied Biosystems, Foster City, CA, USA) with a StepOnePlus thermocycler (Applied Biosystems). All sample values were normalized to 18S rRNA using the 2^−ΔΔCt^ method.

### Hydroxyproline quantitation

Total collagen content deposited within the constructs was determined by measuring hydroxyproline levels. The constructs (5 mm diameter and 2 mm thickness) were homogenized in water by grinding and hydrolyzed using the same volume of 12 N HCl (Fisher, Pittsburgh, PA, USA) at 120°C for 3 h. Hydroxyproline content in constructs was quantitated using a Hydroxyproline Colorimetric Assay Kit (BioVision, San Francisco, CA, USA).

### Histology

After 28 days culture, constructs were removed from the incubator, washed twice, and fixed in buffered paraformaldehyde (4%, Fisher) for 1 day at room temperature. After washing with PBS three times, they were then cryosectioned using a cryostat (CM1520, Leica) at 8 μm thickness. For assessment of GAG deposition, the slides were stained with either Alcian Blue or Safranin O/Fast Green following standard protocols. Images were captured with a CKX41 microscope (Olympus, Japan) equipped with a Leica DFC 3200 camera.

### Immunohistochemistry

Enzymatic antigen retrieval was performed using chondroitinase/hyaluronidase (1 and 5 mg/ml) at 37°C for 30 min, and was suppressed with 1% horse serum (Vector Labs, Burlingame, CA, USA) in PBS for 45 min. After blocking endogenous peroxidase (3% H_2_O_2_ in methanol for 10 min) and non-specific binding [1% horse serum (Vector Labs)], slices were incubated with primary antibodies against collagen type II (Abcam, Cambridge, MA, USA) overnight at 4°C. After washing, biotinylated secondary antibodies (Vector Labs) were applied for 30 min. Staining was developed by treating samples with horseradish peroxidase (HRP)-conjugated streptavidin/NovaRED™ peroxidase substrate (Vector Labs).

### Mechanical property assessment

The mechanical property of constructs at days 0 and 28 were measured using the procedure described before.

### Statistical analysis

All studies were performed with three experimental replicates. Results were expressed as the mean ± SD. Significant differences between control and chondrogenic groups were determined by one-tailed Student’s *t*-test. Significance was considered at *p* < 0.05* and *p* < 0.01**.

## Results

**Figure 1:**
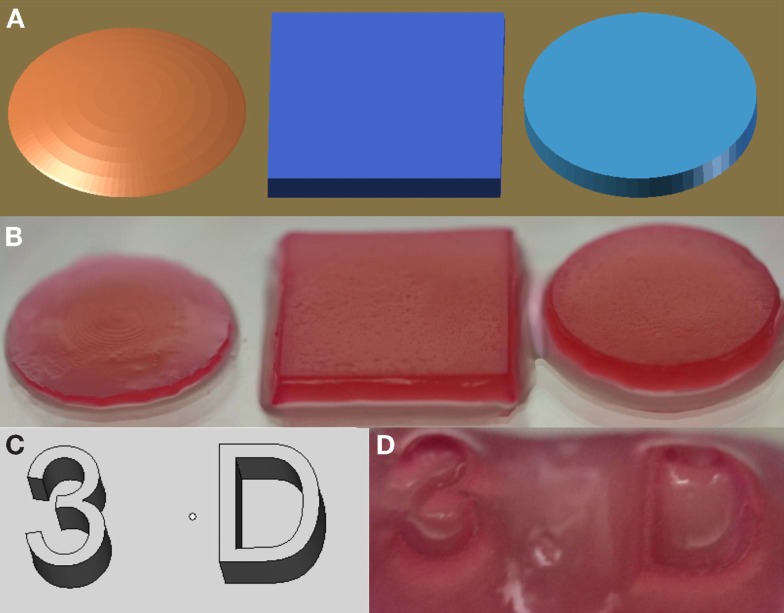
**mPDLLA-PEG constructs generated using VL-PSL**. **(A,B)** PDLLA-PEG hydrogels with spherical, cuboidal, and cylindrical architecture **(B)** based on CAD models **(A)**. **(C,D)** Alphanumeric mPDLLA-PEG hydrogels **(D)** based on CAD models **(C)**. Formulation used includes mPDLLA-PEG (30% w/v), mHA (0.5% w/v), LAP (0.6% w/v), and phenol red (0.025% w/v).

Constructs generated using VL-PSL were first assessed for their ability to faithfully replicate designed architectures. Figure 1 illustrates the construction of various 3D shapes, including conical, cubic, and cylindrical (Figures 1A,B), and more complicated alpha numeric structures (Figures 1C,D). In all cases, the structures produced using VL-PSL mimicked the designs with high fidelity upon visual inspection.

### Degradation analysis

**Figure 2:**
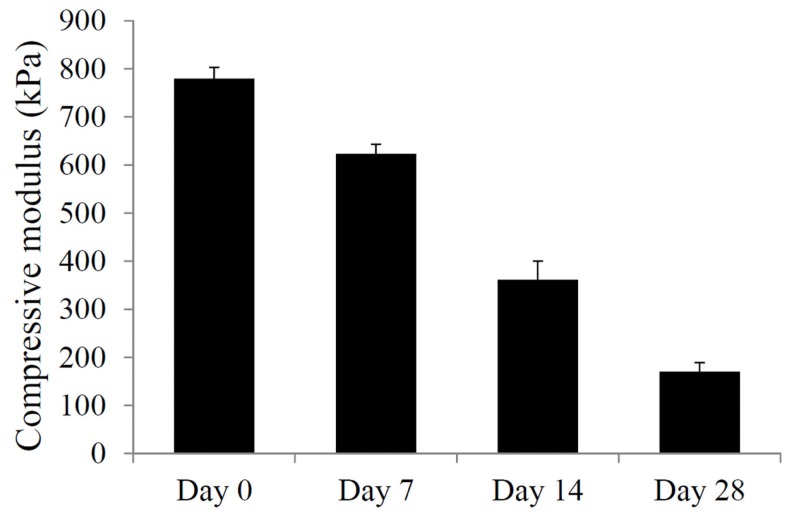
**Mechanical property of mPDLLA-PEG/HA scaffolds incubated in HBSS at 37°C for different times up to 28 days**. Difference between every two groups is statistically significant.

Due to the presence of ester bonds in the mPDLLA-PEG/HA co-polymers, the scaffolds are expected to be degraded through hydrolytic cleavage (Seck et al., 2010). To test the degradation behavior, PSL-fabricated mPDLLA-PEG/HA scaffolds were incubated in PBS at 37°C and their mechanical properties tested at different time points up to 4 weeks. As shown in Figure 2, the compressive modulus of scaffolds significantly decreased with time, demonstrating structural degradation in aqueous solution. After 4 weeks, scaffolds retained only ~25% of their original mechanical strength but still maintained the original cylindrical structure.

### Cell viability assessment

**Figure 3:**
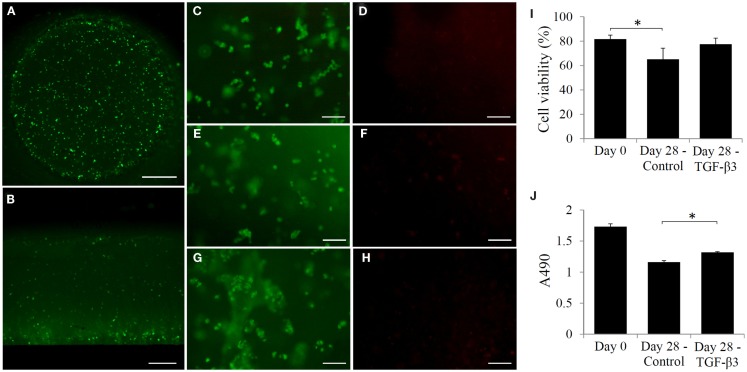
**Cell viability in VL-PSL generated mPDLLA-PEG scaffolds at 0 and 28 days after fabrication**. **(A–C,E,G)** Calcein-AM staining (green, live cells) and **(D,F,H)** EthD-1 staining (red, dead cells) in scaffold demonstrate the cell viability following fabrication throughout VL-PSL method. Cells were seen to be uniformly distributed at different layers. **(I)** Based on live/dead staining results, cell viability at days 0 and 28 in control as well as TGF-β3 group was calculated. **(J)** MTS assay of constructs at days 0 and 28 in control as well as TGF-β3 group. Scale bars: **(A)** 1 mm; **(B)** 500 μm; and **(C–H)** 100 μm. *p < 0.05.

Cell viability was determined immediately after fabrication and also at 28 days after culturing in control and chondrogenic medium. Figure 3A shows a surface view of a Calcein-AM stained construct, and Figure 3B shows a cross-sectional view of the construct at day 0. Uniform distribution of single cells throughout the construct was clearly seen, suggesting that cells remained suspended and separated from each other in the fabrication solution for at least 30 min. Cell viability was determined to be high at 81% after fabrication (Figures 3C,D,I). After 28 days of culture in control medium, cell viability decreased to 65% (Figures 3E,F,I,J). In contrast, chondrogenic medium supplemented with TGF-β3 not only maintained higher cell viability (77%, Figures 3G–I) but also promoted higher overall cell metabolic activity as indicated by MTS assay (Figure 3J). There is a statistically significant difference between the control and the TGF-β3 group in cell number.

### Chondrogenesis analysis

**Figure 4:**
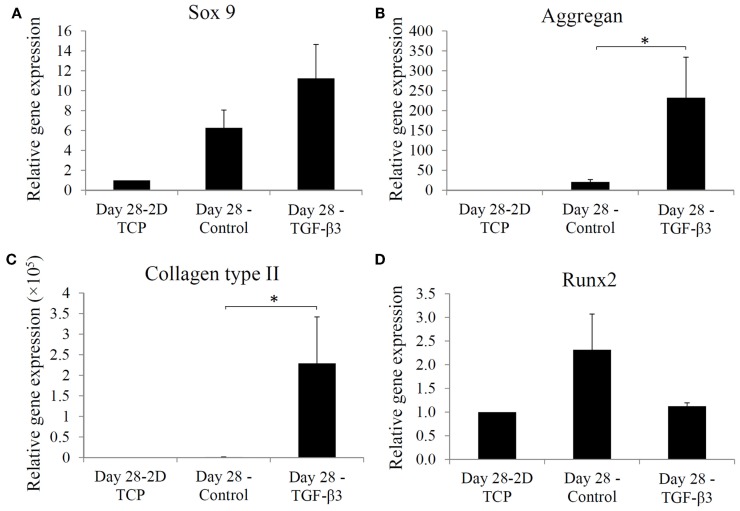
**Real time-PCR analysis of gene expression in hASCs at day 28 in 2D tissue culture plate, control group, and TGF-*β*3 group**. Relative gene expression levels of **(A)** Sox 9, **(B)** aggregan, **(C)** collagen type II, and **(D)** Runx2 at day 28, are normalized to gene expression in 2D culture without chondrogenic induction. *p < 0.05.

Chondrogenic differentiation of the hASCs seeded within the dense mPDLLA-PEG/HA scaffolds and exposed to TGF-β3-containing chondrogenic medium was analyzed by real time RT-PCR for the expression of genes associated with chondrogenesis. Figures 4A–D show the relative levels of gene expression for Sox 9, aggrecan, collagen type II, and Runx2. All three chondrogenic genes, Sox 9, aggrecan, and collagen II, were also considerably higher in the TGF-β3 group. In contrast, Runx2, an osteogenesis marker, expression was found to be higher in the control group. Thus, TGF-β3 chondrogenic medium effectively induced chondrogenesis and concurrently inhibited osteogenesis in hASCs encapsulated within the PSL-fabricated scaffolds.

**Figure 5:**
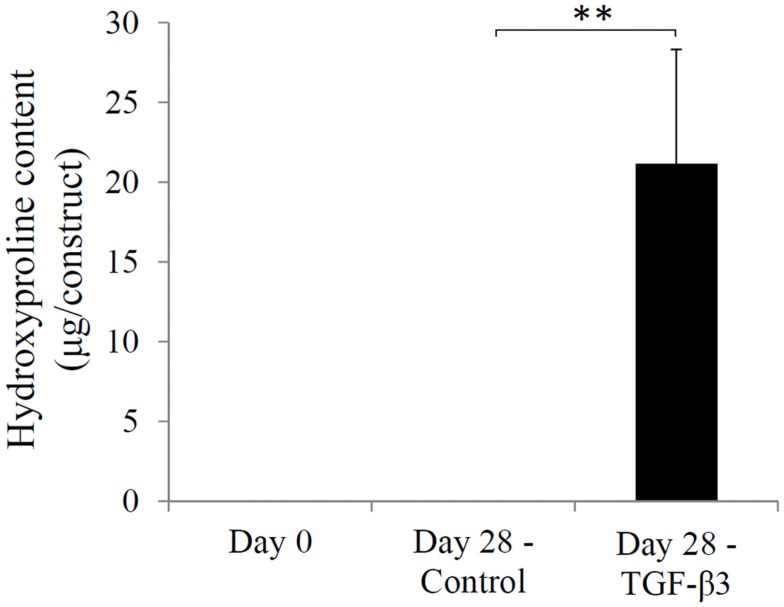
**Hydroxyproline levels in hASC-encapsulated VL-PSL-fabricated constructs in the control and TGF-*β*3 groups at days 0 and 28**. Levels of hydroxyproline in days 0 and 28 control groups were negligible, and levels in the day 28 TGF-β3 group measured at 21.16 ± 7.15 μg/construct. **p < 0.01.

**Figure 6:**
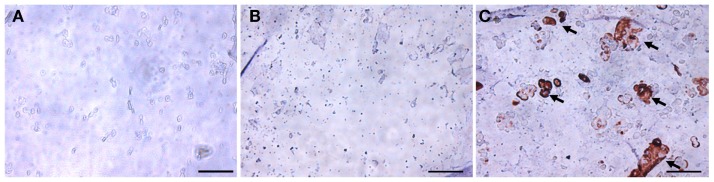
**Immunohistochemical staining for collagen type II in hASC-encapsulated VL-PSL-fabricated constructs**. **(A)** Day 0 immediately after fabrication; **(B)** day 28 control group; and **(C)** day 28 TGF-β3 group. Positive collage type II staining (brown) was only seen in the day 28 TGF-β3 treated group as indicated by arrows. Scale bar: 50 μm.

**Figure 7:**
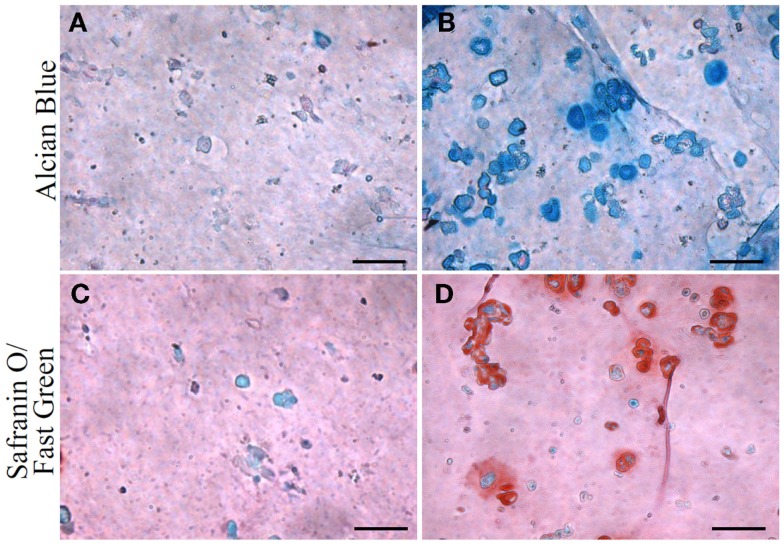
**Glycosaminoglycan (GAG) and proteoglycan content in hASC-encapsulated VL-PSL-fabricated constructs visualized by Alcian blue and safranin O/fast green staining at day 28**. **(A,C)** Alcian blue and safranin O staining, respectively, of control group. Negligible amounts of GAG and proteoglycan are detected. **(B,D)** Alcian blue and safranin O staining, respectively, of TGF-β3 group at day 28, showing strong staining. Scale bar: 50 μm.

The chondrogenic activity of the cells within the constructs cultured in the chondrogenic medium was next assessed based on hydroxyproline assay to estimate the content of newly synthesized collagen. The TGF-β3 group showed a hydroxyproline level of 21.16 ± 7.15 μg/construct versus an undetectable level in the control group (Figure 5). Since this assay did not differentiate between the different collagen types, we further performed immunohistochemistry (IHC) to examine the presence of collage type II. IHC revealed positive staining for collagen type II, a major ECM component in cartilage, only in the TGF-β3 group (Figure 6). GAG and proteoglycan content were also estimated by histological staining with Alcian Blue and Safranin O, respectively. Weak staining was seen in the control group after 28 days culture (Figures 7A,C), while dense, strong staining was seen in the TGF-β3 group (Figures 7B,D). Taken together, these results clearly demonstrate that with TGF-β3 induction, robust chondrogenic differentiation of the hASCs took place within the PSL-fabricated constructs.

**Figure 8:**
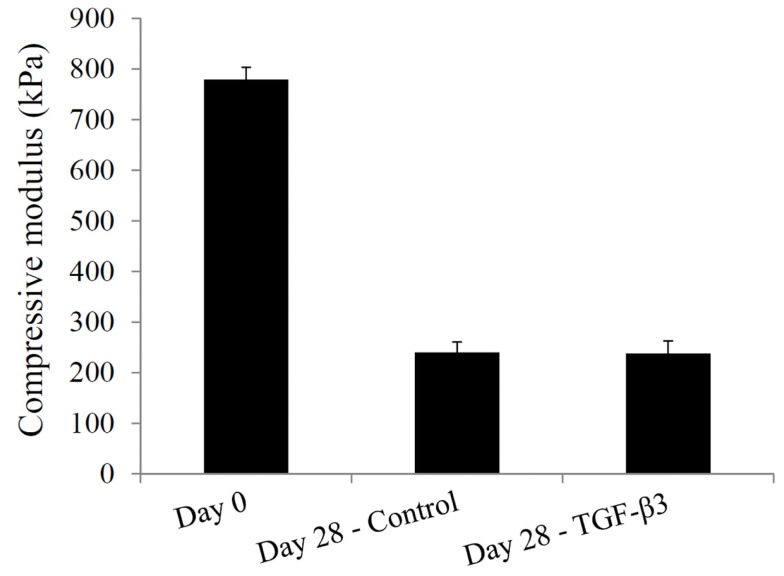
**Compressive moduli of hASC-encapsulated VL-PSL-fabricated constructs measured at culture days 0 and 28 in the control and TGF-β3 groups**.

Lastly, the compressive moduli for the constructs containing the mPDLLA-PEG polymer were measured. At day 0, the compressive modulus was 780 ± 23 kPa, which fell to 240 ± 20 kPa in the control group and 238 ± 25 kPa in the TGF-β3 group by day 28 (Figure 8), principally due to the degradation of the scaffold material.

## Discussion

This study reported the application of a VL-PSL protocol to fabricate live hASC-laden, biodegradable polymeric mPDLLA-PEG/HA constructs with cartilage-like mechanical properties. Within the photocrosslinked constructs, cells were uniformly distributed and underwent robust chondrogenesis upon TGF-β3 stimulation, suggesting the potential application of this technology for cartilage tissue repair.

Tissue engineering has been considered a promising approach to repair degenerated articular cartilage in degenerative joint diseases, such as OA. The goal has been to construct or regenerate tissues that possess the properties of native cartilage, but currently there are many technical challenges. First, articular cartilage is an avascular tissue, which limits the ability of cells of surrounding tissues to infiltrate the defect site following implantation of the engineered tissue implant. Thus, a uniform cell distribution throughout the scaffold that makes up the construct is required so that tissue repair can occur within the entire scaffold and not be confined to the superficial zones. A second challenge is the mechanical environment within the articular joint environment, namely the natural high loads that any scaffold or neo-tissue must withstand. The scaffold thus needs to be inherently mechanically stiff immediately following grafting. A third challenge is that articular cartilage defects, by trauma-induced loss or chronic osteoarthritic lesions, are irregular in shape, which makes moldable cell-seeded materials desirable. No currently available hydrogel materials have completely met these requirements.

Recently, our group introduced a visible-light-based PSL method for live cell-scaffold fabrication. Utilizing this technology, live cells were uniformly distributed within scaffolds with designed architectures. While this presented progress toward the first and third challenges, the chondrogenic potential of constructs was not tested. In addition, the PEG material used is non-biodegradable and does not allow for conversion to native cartilage tissue. Given the limitations of the PEG methacrylate hydrogel, we seek to develop a biomaterial that possesses the ability to be applied in VL-PSL (biocompatibility and water-solubility) as well as exhibits both higher mechanical strength and biodegradability. We have identified PDLLA-PEG as a material that possesses these properties. We then further use high MW HA (>700 kDa) to replace Percoll to maintain hASCs in suspension during fabrication, which not only eliminates the presence of Percoll but also provides native cell-binding ligands. The results in Figure 3B clearly demonstrate that cells remain sufficiently suspended uniformly throughout the scaffold during the 30-min fabrication time.

Even without TGF-β3 stimulation, hASCs cultured in PDLLA-PEG/HA scaffolds showed increased chondrogenic gene expression (Figure 4). Since PDLLA-PEG is an inert material like agarose and alginate, it is reasonable to speculate that this effect resulted from the inclusion of HA and a 3D culture environment (Wu et al., 2010; Schagemann et al., 2013). With TGF-β3 supplementation, hASCs showed robust chondrogenesis indicated by enhanced chondrogenic gene expression and cartilage ECM deposition. Because of the different types of supplemented TGF-β, and variable cell density, culture duration, nature of scaffold, and size of constructs used, no previous data are available for direct comparison to our results. In one study using TGF-β3 treatment for 21 days, collagen type II and aggrecan expression in hASCs encapsulated within fibrin increased 50- and 5-fold, respectively (Park et al., 2011), compared to the 220- and 12-fold increased observed here in PDLLA-PEG/HA, strongly suggesting the chondroinductive property of PDLLA-PEG/HA.

The scaffolds constructed using mPDLLA-PEG/HA are found to rapidly degrade in aqueous solution (Figure 2), which agree with previous report (Seck et al., 2010). Currently, there are relatively limited studies investigating the degradation behavior of this material. Yang et al. reported that half of a PDLLA-PEG (4:1, w/w) scaffold was degraded in 5 days (Yang and Kao, 2006). The relative slow degradation rate of scaffold used in our study might be due to lower PDLLA:PEG ratio and inclusion of photocrosslinkable HA. After 12 weeks of culture in HBSS, the scaffolds still maintain the original architecture (data not shown).

Even with slower degradation rates, the scaffolds lose 75% of their strength in 4 weeks (Figure 2). However, they are still stronger (160 kPa compressive modulus) than commonly used hydrogels, such as agarose, alginate, and gelatin, which have compressive moduli lower than 30 kPa (Byers et al., 2008; Stojkovska et al., 2010; Lin et al., 2014). While this result indicates good mechanical properties of mPDLLA-PEG/HA, it also suggests relatively limited matrix deposition by the encapsulated hASCs (240 kPa compressive modulus at 28 days). Several reasons could account for this limited matrix deposition: (1) cell death during culture, (2) insufficient cell number, and (3) inadequate ECM synthesis. As shown in Figure 3J, cell metabolic activity did decrease after 28 days of culture. In the control group, the cell viability is low (65%), which might be due to limited nutrient exchange through the dense scaffolds, a generic deficiency of hydrogel scaffolds with high mechanical properties. However, the addition of TGF-β3 maintains cell survival (77%). In addition, together with the enhanced levels of gene expression of Sox 9, aggrecan, and collagen type II as well as the presence of collage type II protein, GAGs, and proteoglycan, we conclude that TGF-β3 is able to diffuse into the scaffolds and act to stimulate the encapsulated cells. The observation of metabolic activity reduction in the TGF-β3 group might be due to the relatively low metabolic activity of chondrogenically differentiated hASCs in serum-free environments compared to the naïve hASCs grown in serum-rich conditions without differentiation stimulation (Xu et al., 2008). Therefore, we conclude that the insufficient ECM production in constructs is due to low cell density used during the fabrication process. To optimize deposition of ECM, in future studies, we will use higher initial cell loading density and further optimized chondroinductive conditions. Mauck et al. showed that higher cell density, such as 60 × 10^6^ cells/ml, resulted in considerably higher ECM content than lower cell density culture and contributed to the mechanical property of whole construct (Mauck et al., 2002). Also, it has been reported that the addition of BMP-6 dramatically enhanced the matrix production by hASCs (Puetzer et al., 2010).

In summary, we report here a new combination of biodegradable water-soluble polymers compatible with the VL-PSL fabrication process that has the ability to accommodate seeding as well as chondrogenic differentiation of hASCs and possesses high compressive modulus. Future studies will attempt to: (1) optimize cell density and differentiation conditions to accelerate ECM deposition and (2) address the effect of adding various substituent groups onto the polymer to control its degradation profile, as well as increase and fine-tune the mechanical properties of the scaffold.

## Conclusion

Using the degradable mPDLLA-PEG/HA matrix, we have successfully applied VL-PSL to fabricate scaffolds based on CAD models as the template with high fidelity. hASCs were introduced into the scaffolds during the fabrication process and maintained high viability. TGF-β3-containing chondrogenic medium not only enhanced hASC survival but also effectively induced hASC chondrogenesis, as indicated by increased chondrogenic gene expression and cartilage ECM deposition. Live cell-based PSL-fabricated scaffolds developed in this study thus show great potential in the development of customized repair of cartilage in degenerative joint diseases, such as OA.

## Conflict of Interest Statement

The authors declare that the research was conducted in the absence of any commercial or financial relationships that could be construed as a potential conflict of interest.
